# Pyoderma Gangrenosum Mimicking Necrotizing Fasciitis: A Case Report

**DOI:** 10.7759/cureus.86569

**Published:** 2025-06-22

**Authors:** Takahiro Hase, Akihiro Orita, Takuya Mizukami, Hiroyuki Nakamura

**Affiliations:** 1 Dermatology, Kushiro City General Hospital, Kushiro, JPN

**Keywords:** adalimumab (humira), autoinflammatory disease, necrotizing fascitis, neutrophilic dermatosis, pyoderma gangenosum

## Abstract

Pyoderma gangrenosum (PG) is a rare neutrophilic dermatosis that can mimic other severe conditions, complicating diagnosis. We report a case of PG of the knee initially mistaken for necrotizing fasciitis (NF) in a 68-year-old woman who presented with knee swelling, erythema, papules, and pustules following a fall. Despite antibiotic therapy and surgical debridement for suspected NF, the patient’s fever persisted, and laboratory markers (elevated WBC and C-reactive protein) did not improve. Histopathology revealed a dense neutrophilic infiltrate, and negative cultures ruled out infection, leading to a PG diagnosis. Treatment with oral prednisolone (60 mg/day) rapidly resolved symptoms, followed by negative pressure wound therapy, skin grafting, and adalimumab initiation. The patient stabilized without relapse. Key diagnostic clues included abundant pustules, characteristic of pustular PG, and the absence of fat tissue necrosis during surgery. This case highlights the diagnostic challenge of distinguishing PG from NF, as both may involve deep tissue inflammation. While histopathology and cultures are critical, results are delayed, necessitating reliance on clinical observations. Careful assessment of skin lesions and evaluation of fat necrosis (finger test) can prevent unnecessary surgical interventions, emphasizing the importance of considering PG in atypical presentations of suspected NF.

## Introduction

Pyoderma gangrenosum (PG) is a rare neutrophilic dermatosis with an estimated incidence of 3-10 cases per million per year [1]. It most commonly presents as painful ulcerative lesions on the lower extremities, gluteal region, or abdomen, though atypical sites and presentations are well-documented. While the ulcerative form is the most frequent clinical manifestation, PG encompasses several variants, including pustular, bullous, and vegetative types [1,2].

The exact etiology of PG remains poorly understood, but it is frequently associated with systemic conditions such as inflammatory bowel disease, hematologic malignancies, or rheumatoid arthritis [1,2]. A hallmark of PG is its propensity for exacerbation following minor trauma, a phenomenon known as pathergy [1,2]. The heterogeneous clinical presentation of PG often mimics severe infections, such as necrotizing fasciitis (NF), posing significant diagnostic challenges that can lead to delayed or inappropriate treatment.

Misdiagnosis may result in unnecessary surgical interventions, which can exacerbate PG due to its pathergic nature. We report a case of PG involving the knee, initially mistaken for NF, to highlight the diagnostic pitfalls and clinical nuances of this rare condition.

## Case presentation

A 68-year-old woman presented with swelling of the left knee two weeks after a fall. Notably, the swelling had developed one week prior to her presentation (one week after the fall). The affected area was associated with severe tenderness and pain, despite the patient's regular use of non-steroidal anti-inflammatory drugs (NSAIDs) for chronic back pain.

**Figure 1:**
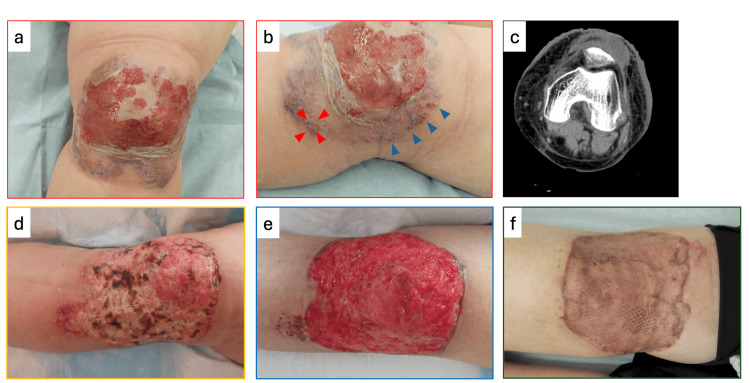
Clinical photographs _(a, b) Initial presentation at our hospital, showing an erosive ulcer with vesicle formation and knee swelling. Pustules were observed at the lesion periphery (red and blue arrowheads)._ _(c) Computed tomography at initial presentation revealed hyperintensity in the subcutaneous fat tissue without evidence of fluid collection._ _(d) Post-surgical debridement; fever and laboratory data remained unchanged. Oral prednisolone (60 mg/day) was initiated on postoperative day 4 (POD4), with subsequent rapid improvement in symptoms and markers._ _(e) Skin ulcer flattened following negative pressure wound therapy (RENASYS®; Smith & Nephew plc, London, United Kingdom)._ _(f) Successful split-thickness skin graft performed without complications. Adalimumab (ADA) 160 mg was initiated to prevent relapse and facilitate prednisolone tapering._

Physical examination revealed marked erythema and swelling of the knee, with a 70 × 50 mm erosion on the extensor surface (Figure 1a). Pustular formation was noted at the periphery of the lesion (Figure 1b). Computed tomography (CT) revealed subcutaneous fat stranding without evidence of fracture or significant fluid accumulation in the subcutaneous tissue or joint space (Figure 1c). Laboratory findings included leukocytosis (38,300/mm³) and elevated C-reactive protein (20.16 mg/dL). The Laboratory Risk Indicator for Necrotizing Fasciitis (LRINEC) score was calculated to be 8. Suspecting a bacterial infection, we initiated broad-spectrum antibiotics (meropenem, vancomycin, and clindamycin), but symptoms persisted.

**Figure 2:**
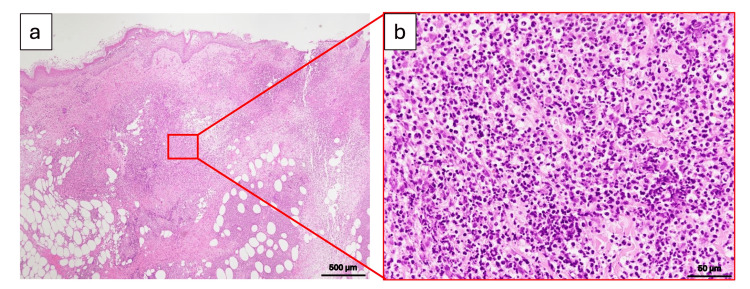
Histopathological findings from a pustule at the lesion periphery _(a) Low-power view (H&E staining, scale bar: 500 μm) showing abundant inflammatory cell infiltration from the mid-dermis to deep dermis, accompanied by fat necrosis._ _(b) High-power view (H&E staining, scale bar: 50 μm) revealing that the infiltrated cells are predominantly neutrophils._

Given the clinical presentation, knee swelling accompanied by erosion, and markedly elevated inflammatory markers, along with a high LRINEC score of 8, NF was strongly suspected. Surgical debridement was performed on hospital day 2. During the operation, dishwater-like fluid, a characteristic of NF, was not observed, and the subcutaneous fat tissue was well preserved without evidence of necrosis. Based on these observations, we suspected the possibility of another diagnosis, including PG, and performed a skin biopsy during the procedure (Figure 1b, red arrowheads). Histopathology revealed a dense neutrophilic infiltrate (Figure 2), and blood and wound cultures were negative.

Despite surgery, the patient remained febrile, with unchanged laboratory markers; the surgical ulcer showed no improvement (Figure 1d). Based on clinical and histopathological findings, we diagnosed PG and initiated oral prednisolone (60 mg/day, 1 mg/kg/day) on postoperative day 4.

Fever resolved rapidly, and laboratory markers improved. There was no relapse of PG despite tapering prednisolone by 10 mg every two weeks. A thorough evaluation for underlying conditions associated with PG was performed; however, laboratory findings did not indicate any hematologic disorders, and the patient exhibited no clinical signs suggestive of inflammatory bowel disease. Despite the tapering of prednisolone, the surgical ulcer remained unchanged for more than four weeks.

For the treatment of the ulcer, negative pressure wound therapy (RENASYS®; Smith & Nephew plc, London, United Kingdom) was applied for two weeks until it flattened (Figure 1e), followed by a split-thickness skin graft while the patient was on prednisolone 30 mg/day. The skin graft was successfully performed without recurrence of PG, and prednisolone was tapered by 5 mg every two weeks (Figure 1f). Adalimumab was started to prevent relapse and facilitate prednisolone tapering. The patient was discharged after rehabilitation and remains relapse-free.

**Figure 3:**
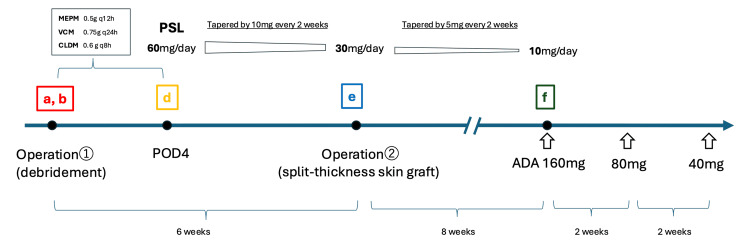
Timeline of clinical interventions and treatment ^(a, b) initial presentation; (d) debridement and postoperative day 4 prednisolone start; (e) negative pressure therapy; (f) split-thickness skin graft and ADA 160 mg initiation, followed by ADA 80 mg, and maintained with 40 mg every two weeks; prednisolone tapered from 60 mg/day to 30 mg/day (by 10 mg every two weeks), then to 10 mg/day (by 5 mg every two weeks).^ ^ADA: adalimumab; MEPM: mouse embryonic palatal mesenchyme; VCM: vancomycin; CLDM: clindamycin; PSL: prednisolone^

The treatment course is outlined in Figure 3.

## Discussion

Distinguishing PG from NF is challenging due to overlapping clinical features. NF is a rapidly progressive bacterial infection requiring urgent surgical intervention, whereas PG is a sterile inflammatory condition that may worsen with surgery due to pathergy. Bisarya et al. outline key differences: (i) PG typically involves the epidermis to the dermis, whereas NF extends to deeper tissues, including fascia or muscle (assessable via the finger test); (ii) PG is often associated with systemic diseases (e.g., inflammatory bowel disease, hematologic disorders), while NF is linked to patients with uncontrolled diabetes or immunosuppressive conditions, where these conditions poses a risk for developing NF; (iii) PG worsens with surgical intervention (pathergy), unlike NF; (iv) PG shows negative cultures, while NF yields positive bacterial cultures; and (v) PG typically follows a gradual clinical course, unlike NF’s rapid progression [3]. 

Differentiating PG from NF is challenging due to overlapping clinical features, yet critical, as NF requires urgent surgical intervention. Medical imaging, such as CT or magnetic resonance imaging (MRI), is commonly used to assess deep tissue involvement. Imaging helps distinguish NF, characterized by deep fascial inflammation and fluid collection, from conditions like cellulitis. However, imaging findings for PG are poorly documented, with only two reported cases [4,5]. Park et al. reported a case of PG mimicking NF, where MRI showed fascial inflammation and fluid collection, initially suggesting NF [5]. Although NF typically exhibits more extensive deep tissue inflammation than PG, CT, and MRI cannot reliably differentiate the two, as both may present similar deep tissue changes, as seen in our case’s CT findings. 

Ultrasonography has been reported to be a valuable tool in the early diagnosis of NF. In particular, the detection of fluid accumulation along the fascial planes is considered a sensitive indicator suggestive of NF [6].

In this case, two key observations facilitated the diagnostic process: (i) the presence of erythematous papules and pustules at the periphery of the lesion, an uncommon finding in NF, and (ii) intraoperative findings of well-preserved subcutaneous fat tissue without evidence of necrosis, which suggested an alternative diagnosis.

## Conclusions

PG exhibits diverse clinical presentations, which can complicate its diagnosis. In the present case, knee swelling and erosion formation initially mimicked NF, underscoring the lack of definitive laboratory or clinical markers to reliably differentiate PG from NF. The absence of subcutaneous fat necrosis observed during surgery served as a critical diagnostic clue that raised suspicion of PG.

Although the "finger test" was not performed in this case, its application at the bedside may have contributed to an earlier and more accurate diagnosis. This case highlights the importance of comprehensive clinical assessment and detailed medical history, as laboratory findings alone may be inconclusive. Such vigilance is particularly vital to avoid unnecessary surgical interventions, which may aggravate PG due to its pathergic nature.
